# Dielectrophoresis for Biomedical Sciences Applications: A Review

**DOI:** 10.3390/s17030449

**Published:** 2017-02-24

**Authors:** Nurhaslina Abd Rahman, Fatimah Ibrahim, Bashar Yafouz

**Affiliations:** 1 Department of Biomedical Engineering, Faculty of Engineering, University of Malaya, 50603 Kuala Lumpur, Malaysia; haslin42@siswa.um.edu.my (N.A.R.); bashar.yafouz@um.edu.my (B.Y.); 2 Centre for Innovation in Medical Engineering (CIME), Faculty of Engineering, University of Malaya, 50603 Kuala Lumpur, Malaysia; 3 Faculty of Engineering and Information Technology, Taiz University, 6803 Taiz, Yemen

**Keywords:** dielectrophoresis, biomedical sciences, diagnostic, point-of-care

## Abstract

Dielectrophoresis (DEP) is a label-free, accurate, fast, low-cost diagnostic technique that uses the principles of polarization and the motion of bioparticles in applied electric fields. This technique has been proven to be beneficial in various fields, including environmental research, polymer research, biosensors, microfluidics, medicine and diagnostics. Biomedical science research is one of the major research areas that could potentially benefit from DEP technology for diverse applications. Nevertheless, many medical science research investigations have yet to benefit from the possibilities offered by DEP. This paper critically reviews the fundamentals, recent progress, current challenges, future directions and potential applications of research investigations in the medical sciences utilizing DEP technique. This review will also act as a guide and reference for medical researchers and scientists to explore and utilize the DEP technique in their research fields.

## 1. Introduction

The need for the rapid detection of diseases is becoming crucial to prevent the loss of life. It is often too late to take action if the disease is at the final stage. The patients’ chances to live and be cured increase if the disease diagnosis is performed as early as possible [1]. There are many factors that can cause diseases, such as autoimmune disorders, disruption of the balance in the human body system, cancers, bacteria, viruses, and fungal and other microorganism infections [2,3,4]. During the disease process, there are certain complex changes that occur in cells ranging from the molecular integrity to cell morphology [5]. There are various schemes used to detect diseases, including the use of dye marker labelling, enzyme-base labelling assays and nucleic acid-based assays [6,7,8,9,10]. All of these methods form the basis for nearly all of the pathology laboratory tests in hospitals. Certain assays use fluorescence substances to label the antibodies and antigens to quantify them. Some use the antibody to label the antigen [11]. All of these methods feature high sensitivity and specificity values; however, the procedures of conducting these tests often require trained clinical personnel and expensive equipment [12]. Furthermore, while patients are on the verge of the right treatment, these tests are time consuming, which could be a major drawback of these techniques [13]. Economically, these tests are considered expensive to be run in small laboratories, typically located in small villages; thus, there is the need send the samples to a central laboratory. This procedure takes time and causes a delay in the treatment given to the patients. In addition, any contaminants or analytical errors during the process will lead to false-negative results, placing technicians in charge of handling techniques at a disadvantage [14].

A fast and accurate disease diagnosis is the best first choice to save human life. This option cannot be achieved without an in-depth understanding of the physiology of living cells [15,16]. Distinguishing the properties between a healthy and pathological state of cells has become the major interest of study to researchers to unravel the mystery of the disease and deliver the unmet rapid disease treatment [17]. Recently, a few researchers have directed their efforts to distinguish between healthy and infected/unhealthy cells utilizing a technique called dielectrophoresis (DEP) [18,19,20]. DEP is the movement of particles by a trapping force in a non-uniform electric field when the particles and surrounding medium have different polarizabilities. The polarization of the charged or neutral particles is induced by an electric field generated from alternating current (AC) or direct current (DC) potentials. The polarized particles would array in various motions, including attraction or repulsion from the electrode by changing the frequency of the applied electric field, and this motion is in response to positive DEP (p-DEP) or negative DEP (n-DEP), respectively. These fundamentals were officially named “*dielectrophoresis*” by Dr. Herbert A. Pohl, a scientist at the Naval Research Laboratory at Anacosta (DC, USA) [21]. In Phol’s paper, he defined DEP as a phenomenon seen in the relative motion of suspensions and media resulting from polarization forces produced by an inhomogeneous electric field. Since then, DEP research has expanded into various fields in the industry, including microfluidics [22,23], biosensors [24,25], environmental studies [26,27] and medical diagnostics [28,29].

This paper reviews the present studies and applications of DEP in the medical science field. The paper starts with a brief explanation of the DEP background, theory and its advantages. Next, the previous DEP investigations in medical sciences are reviewed including: eukaryotic and prokaryotic cells, oncology, stem cells, drug delivery, virus, bacteria, microorganism, fungi, DNA, proteins and enzymes. Finally, DEP current challenges and limitations are highlighted along with the potential future applications that can be conducted using DEP in the medical science research field.

## 2. DEP Background

### 2.1. Dielectrophoresis (DEP) and Electrophoresis (EP)

Many confused on the terms of DEP and EP. DEP technique manipulates particles in non-uniform electric field, while in electrophoresis the particles respond to the uniform direct current (DC) voltage to energize the electrode and attract particles. The movement of particles in DEP is based on the difference in polarizability between the particles and the surrounding medium [30]. If the particles move towards the electrode edge, the region of high electric field gradient, then the response is called positive DEP (p-DEP), while if the particles move away of the electrode edge, then the response is called negative DEP (n-DEP) [31]. In DEP the particles itself carry electrical potential, and respond uniquely to the different frequencies. On the contrary, the particles manipulation in the electrophoresis technique is controlled by the particle size, density, molecular weight and purity. Electrophoresis of positive charged particles (cations) is called cataphoresis, while electrophoresis of negatively charged particles (anions) is called anaphoresis. Another difference between the two technique is that DEP technique can create a trap of particles using electromagnetic fields [32], while electrophoresis cannot create stable non-contact traps of particles.

### 2.2. DEP Theory

**Figure 1:**
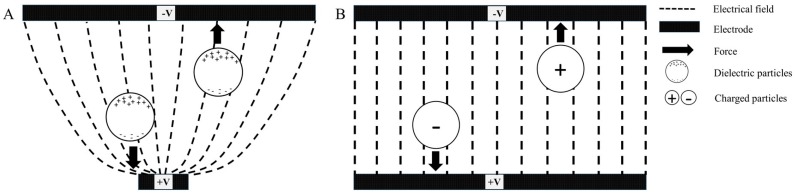
Schematic diagram of DEP vs. EP. (**A**) shows p-DEP and n-DEP effects where the dielectric particles move towards the high and low electric field gradient, respectively; (**B**) shows EP in which cations and anions move towards negative and positive electrode, respectively.

DEP force is initiated by applying a non-uniform AC electrical field that manipulates the motion of particles by creating polarisability gradient between the particles and the suspending medium. DEP technique is exploiting the mechanical and electrical properties of the cells and the binding properties of the proteins and cell surface for the uniquely induce cell motion. When cells/particles are exposed to this non-uniform electric field, two different forces occur between the cells and surrounding medium leading to a resultant force. The motion of the cells/particles can be in response to pDEP or nDEP effects depending on the relative polarisability between the cell and suspending medium. Figure 1 shows the illustration of DEP phenomena, in which p-DEP effect occurs when the cell travels toward high electric field gradient, while n-DEP effect occurs when the cells travels toward the low electric field gradient, both of this phenomena depends on Clausius-Mossotti (CM) factor.

DEP force applied to homogeneous sphere of a radius *r* in a suspension medium of a relative permittivity *ε_m_* can be demonstrated by:

(1) 〈F→DEP〉=2πr3εoεmRe[K(ω)]∇E2

where *ω* is the angular frequency of the applied field, ∇*E* denotes the electric field gradient; and Re[*K*(*ω*)] is the real part of the CM factor. According to this formula, DEP force is controlled by the CM factor which is frequency-dependent. CM factor (*K*(*ω*)) is expressed as $\frac{\varepsilon_{p}^{*}-\varepsilon_{m}^{*}}{\varepsilon_{p}^{*}+2\varepsilon_{m}^{*}}$, where $\varepsilon_{p}^{*}$ is the complex permittivity of particles and $\varepsilon_{m}^{*}$ is the complex permittivity of medium. The complex permittivity $\varepsilon^{*}$ is given by $\varepsilon -j\frac{\sigma }{\omega }$, where $j=\sqrt{-1}$, *σ* is the material conductivity and *ω* is the angular frequency. The direction of the DEP force is independent of the applied voltage; which means changing the voltage would not interfere with the direction of the resultant DEP force. However, the relative polarisability of the cells and the suspending medium can be manipulated by controlling the frequency of the applied electric field. The DEP force becomes zero at a specific frequency and the particles do not move. This specific frequency is known as the *crossover frequency* or *zero force frequency* [33]. This phenomenon takes place when the real part of the effective polarisabilities of the particle and the surrounding medium equal each other (i.e., Re[*K*(*ω*)] = 0).

**Figure 2:**
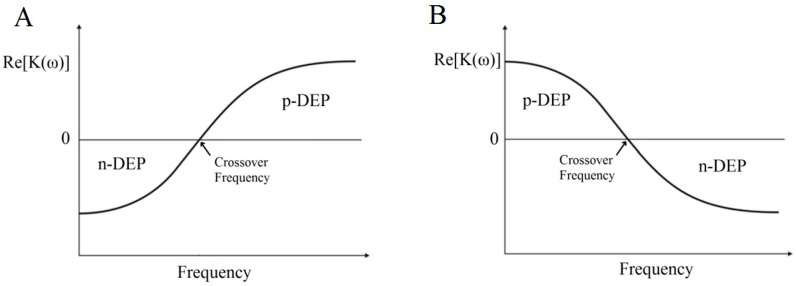
DEP spectrum (Re[*K*(*ω*)] vs. frequency) of a polarizable particle. (**A**) when *σ_p_* < *σ_m_* and *ε_p_* > *ε_m_*; (**B**) when *σ_p_* > *σ_m_* and *ε_p_* < *ε_m_*.

By studying carefully CM factor, it was established that the conductivity controls the low frequency DEP behavior, while the permittivity controls the high frequency behavior [34]. Therefore, there are two main cases that govern the relationship between applied signal frequency and Re[*K*(*ω*)]. The first case occurs when *σ_p_* < *σ_m_* and *ε_p_* > *ε_m_*; making Re[*K*(*ω*)] negative at low frequencies and positive at high frequencies. On the other hand, the second case is when *σ_p_* > *σ_m_* and *ε_p_* < *ε_m_*, then Re[*K*(*ω*)] becomes positive at low frequencies and negative at high frequencies [34]. Figure 2 illustrates the relationship between the applied signal frequency and Re[*K*(*ω*)] with respect to the particle and surrounding medium permittivity and conductivity.

### 2.3. Electrode Geometry

The non-uniform electromagnetic fields needed to generate the DEP forces are created by microelectrodes patterned using various microfabrication methods. A number of techniques exist for the fabrication of microelectrodes, including wet etching, reactive ion etching, conventional machining, soft lithography, hot embossing, injection molding, laser ablation, in situ construction, and plasma etching [35], however, photolithography is considered the basis for most of these processes. Each microelectrode geometry is designed to investigate specific research purpose. Electrode geometry is important factor to ensure stable and sufficient DEP forces being applied to the induced particles. Since the DEP technique directly affects cell physiology, several electro-physiological effects need to be considered when designing the electrode geometry. Factors such as overheating (Joule heating) by the electric field may cause dehydration, membrane disruption and death to the cells. Table 1 summarizes the electrode geometries used in DEP research and their applications. It can be concluded that there is no specific electrode geometry can occupy all the research applications.

iDEP is a particle confining technique based on the movement of matter in inhomogeneous electric fields that employs insulating structures embedded in a microchannel to produce electric field gradients [36,37,38,39]. The inhomogeneous electric field required for iDEP platforms is induced when the cross-sectional area of the microchannel is “pinched” by the presence of electrically insulating structures between external electrodes [38]. It has been reported that the polarization of the post (obstacles) depends on the ratio of the medium’s and the post’s permittivity [36]. The magnitude of the polarization is increasing with deviation of the permittivity ratio from unity. The optimization of the microchannel structures with arrays of posts in iDEP can result in single particle trapping. Furthermore, it has been demonstrated that when lowering the depth of the highly constricted channels to submicron sizes, the degree of Joule heating will substantially be reduced [37]. This will widen the range of voltages and media conductivities that can be applied to achieve rapid enrichment of target particles by DEP. While in [38], the iDEP showed that particles’ size and the shape of microelectrode have significant effects on the magnitude, location, and shape of the DEP trapping regions. On the other hand, in [39] the researchers were able to segregate certain bio-particles by using asymmetric shaped insulating posts coupled with low-frequency electric potentials. Moreover, the electrodes in [40] were fabricated onto quartz substrates using photolithography technique and were used for the direct mapping of the suspended particles’ spatial concentrations. Whereas in [41], rectangular electrodes that can be selectively and sequentially activated were utilized to provide sufficient DEP force to manipulate liquids by modulating the input frequency.

The cylindrical electrodes in [42] were to immobilize proteins. The combination of alternating electric fields with nanometer-sized electrodes allowed the permanent immobilization of proteins by DEP force. Furthermore, yeast individual characterization was reported by applying different consecutive frequencies [43]. The electrodes precisely controlled the translational movement of microscopic particles by DEP for the determination of the cells’ electrical properties.

Interdigitated electrode is the most common geometry used in DEP. In [44], the cells were characterized by measuring the DEP force by varying the applied frequencies. While the quantification in [45] was done by the calculating the nanoparticles density variations by monitoring the distribution of the frame pixel intensities. Moreover, researchers in [46] have replaced the common interdigitated electrode plate by cylindrical interdigitated electrode array to avoid the Joule heating and reduce power consumption.

The microarray dot electrode used in [18], was chosen because of the confined area for the DEP manipulation. Induced cells were either collected at the dot center in the case of n-DEP, or travel toward the dot edge in the case of p-DEP. The light intensity shifts in the central region of the dot were analyzed, and the DEP spectrum of the induced cells was plotted. While in [47], the circular electrode provided high separation efficiency without enlarging the device size. The particle separation method is based on non-uniform circular travelling-wave electroosmosis (TWEO). This technique causes an interaction between the electric double layer charge and the tangential electric field on the electrode; leads to fluid flow in the direction of the wave.

## 3. DEP Applications in Biomedical Sciences

Medical sciences are an integrated multidisciplinary field of study that has mainly focused on healthcare diagnostics [48,49,50]. It emphasizes the nature of human health and diseases and attempts to achieve unmet medical needs. Many techniques and disciplines have been implemented to help medical researchers and scientists to diagnose and assess diseases and patient medical conditions [51,52,53,54]. Research in the medical field requires in depth understanding of multidisciplinary fields with more innovative research programs to develop better solutions for healthcare problems [55]. To support research needs, major financial pillars become one of the grave factors to sustain research development. However, financial support is difficult to be secure in villages, rural areas and developing countries. Therefore, well-developed diagnostic techniques that can operate with minimal laboratory infrastructure are crucially required to deliver the necessary medical need to the population in developing countries [56].

Bioelectric signals from cells have been proven to carry various useful information about the cell status [57,58]. There many sources of bioelectric signals of cells; one is the sodium potassium pump in the membrane cell matrix. The movements of the sodium and potassium ions through the cell membrane create membrane excitability and an electrical gradient due to the different charges inside and outside the cell membrane matrix [59,60,61,62]. The cell patterning, effect of chemical analytes, and cell-to-cell interactions with the extracellular matrix can be determined by exploiting the dielectric properties of the cells without the need for any tags or labels. In the following subsection, the DEP applications in biomedical sciences are critically reviewed.

### 3.1. Cells

#### 3.1.1. Eukaryotes and Prokaryotes

Cells can generally be categorized as eukaryotic and prokaryotic. This kingdom classification is based on the differences in the cytology and molecular structures of the cells. The most distinct different features between eukaryotes and prokaryotes cells are the presence of a membrane-bound nucleus in eukaryotes [63]. Eukaryotic cells have complex membrane-bound organelles such as the endoplasmic reticulum, lysosomes and peroxisomes, microtubules, mitochondria, and histones for DNA wrapping. Meanwhile, prokaryotes possess much simpler cell structures with a smaller ribosome size and single circular chromosomes [64,65]. An example of eukaryotic cells is human cells, while bacteria are considered prokaryotic cells. Table 2 summarizes the previous investigations conducted on eukaryotic and prokaryotic cells using the DEP technique.

DEP techniques are mainly being used in eukaryotic and prokaryotic cells. The electrophysiology of the cell is being manipulated to serve different purposes such as trapping, sorting, isolation and separation of cells or microorganisms. Since DEP technique enjoys certain features including label free, fast and inexpensive, DEP has the potential to be implemented in the future diagnostic techniques that can be applied in daily medical laboratory routines. Techniques such as immunohistochemistry that need antibody and fluorescence labelling do have high sensitivity and specificity; however, for their operation, a specific antibody, a fluorescent dye, and a dark-field microscope are needed. Furthermore, sometimes, due to toxin reactions, cell degradation and the fluorescence dye fading during the washing step may lead to false-negative results [68,69,70]. These problems can be countered using DEP techniques, which are label free.

#### 3.1.2. Cell Membrane

**Figure 3:**
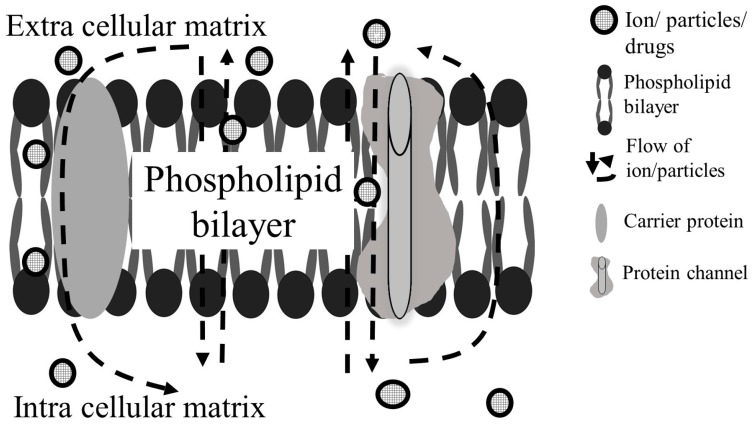
Plasma membrane with the ion/particle flow into and out of the cells. Certain ion/particles can simply pass through the plasma membrane by diffusion, while others need to pass through the protein channel or carrier protein.

The cell membrane is also known as the plasma membrane; in eukaryotic cells, the membrane is selectively permeable, enabling only certain compounds to pass through the membrane layer. It acts as a controller for the movements of ions and molecules in and outside of the cells. The cell membrane structure is made of a phospholipid bilayer (see Figure 3), while the proteins are bound to the membrane surface and within the membrane layer. Proteins on the extracellular membrane surface are involved in cell-to-cell interactions, whereas the proteins located on the intercellular membrane are responsible for the structures that build the protein channels and protein pores. Some small molecules and ions can just diffuse through the plasma membrane, and some ions and molecules can just move in or outside the cells by osmosis. However, for some large molecules, active transport is needed to control molecular movement across the cell membrane. Active transport allows the molecules to move against the concentration gradient, consuming energy [71,72,73].

The membrane potential, or transmembrane potential, is the difference in the electrical gradient between the intracellular matrix and extracellular matrix, the potential gradient is caused by the ionic imbalance on both sides in or outside the plasma membrane. The DEP response of a cell is mostly determined by the physicochemical properties of the plasma membrane. When an external electric field is applied, ions are transferred from the external and internal surfaces of the plasma membrane; subsequently, the charges will join together to produce dipole movement [74].

Little research has been conducted on the plasma membrane using the DEP technique. In 2013, Abdallah et al. [75] sorted crystallized membrane proteins by combining microfluidics and microbeads for membrane protein structure studies. The study of cell membranes is highly important in biomedical research. It can configure the functional access in cell membranes, for example the ion channel activity in membrane surfaces, and which is an important prerequisite in drug discovery studies [76].

### 3.2. Oncology Research

Oncology is the study targeted on the diagnosis and treatment of cancer. The aim of oncology research is to detect cancer as early as possible through screening and to identify the cancer stages. Cancer is an abnormal malignant cell growth that has metastasized and travelled to nearby tissues or other parts of the body invading organs and systems [77]. On the other hand, the treatments are multilevel and multimodal depending on the cancer type and stage. Oncology also includes the management of the side effects of treatment [78]. The critical challenges of oncology research include the isolation of rare cancer cells from complex samples (i.e., blood), confirmation and differentiation of cancer type, and monitoring the treatment progress by assessing the cell morphology. As the DEP technique can differentiate the healthy and pathological states of cells by exploiting cell dielectric properties, DEP has been used in several oncology investigations.

Different cells have different sizes, surface area and morphology. The main parameters that govern the DEP cell separations are attributed to the unique differences of cell electrical properties “*fingerprints*”. Each cell at a certain pathological state feature a certain crossover frequency which can be used for the characterizations of infected cells [79].

Despite DEP crossover frequency, the ionic conductivity of the suspending medium also has a major effect on the DEP cell differentiation. A particular medium conductivity, can be adjusted in a very narrow AC frequency band [19,79]. Table 3 reviews the research in oncology using the DEP technique.

Cancer cells are hard to detect and isolate from normal cells, because genes are mutated and differentiated into subsets, and a specific biomarker is needed for their detection [83]. Sequencing, cell sorting and the use of flow cytometry in cancer detection require highly trained workers and expensive equipment at a high cost burden [84,85,86].

According to Table 3, DEP is fast and label-free technique and can improve the screening performance of cancer cells when combined with other devices (i.e., microfluidic platforms). For example in [79], the interdigitated electrode is combined with DEP field flow fractionation (DEP-FFF) allowing high throughput cells isolation and characterization. The integrated techniques also enhanced the cell recovery for future clinical analysis. Therefore, DEP can be used to reduce the complexity of the current techniques for cancer cell identification.

### 3.3. Stem Cells

Stem cells are a class of undifferentiated cells that can differentiate into specialized cell types. It can be found in embryos (embryonic stem cells), during the blastocyst phase of embryological development and in adult tissue [87,88,89]. Adult stem cells, also called somatic stem cells, are undifferentiated cells found among differentiated cells in a tissue or organ. The primary roles of adult stem cells are to maintain and repair the tissues in the body [88]. Stem cells are considered the top of the hierarchy of cells, and they generate progenitor cells (immature cells) in which the differentiation and proliferation are restricted until they become mature specialized cells [89,90]. The condition of being undifferentiated, mitotically active precursor cells enables the manipulation of stem cells to pose distinct developmental potential and alternative growth [91]. 

Stem cells have become a trend of research because of their ability to differentiate, and either remain as a stem cell or become specialized function cells such as muscle cells, red blood cells, and liver cells. Stem cells can be used in the treatment of wound healing, cell therapy, and drug therapies and have the potential to be developed into artificial organs [92,93,94,95]. Few researchers have taken advantage of DEP technology to assist them in the isolation and separation of stem cells. Table 4 summarizes recent DEP studies on stem cells.

In stem cell research, the differentiation, fractionation, isolation and sorting of cells is very crucial. Stem cells need to be differentiated from the progenitors, the lineages and from other progenies cells. Commercialized cell sorter systems such as Fluorescent Activated Cell Sorting (FACS) [101] and cell labeling [102] require considerable tedious cell preparation, whereas DEP is a simple and cost-effective technique that can be implemented in this area. Recently, the scientists have demonstrated a consistent link between membrane capacitance and the cells’ electrophysiological properties by using DEP. DEP have been able to distinguish the progenitors and the lineage cell that defines progenitors during the cell differentiations [96,97]. In [96] the castellated electrode arrays based on a microfluidic DEP-assisted cell sorting (DACS) have differentiated and enriched the stem cells by applying different frequencies. While the design in [97] is based on oblique interdigitated electrodes and used to sort and differentiate stem cells and enhanced the efficiency of cell recovery and collection. The stem cells differentiation is not merely limited to the progenitor and lineage differentiation; however when DEP is integrated with stereolithography (SL) technique, the three dimensional cell control encapsulated in hydrogel was able to organize the cell patterning; resembling the actual in vivo cell environment compared to conventional two dimensional tissue culture method [98]. Furthermore, hybrid DEP-FFF platforms have been used for stem cells fractionation utilizing interdigitated electrodes. It provides rapid, label-free progenitor cell enriched tissue fractions for on-demand tissue preparation in the clinical practices [99].

DEP is a powerful technique to monitor, interrogating, characterizing, trapping and sorting the stem cells even in mixed cultures. The DEP technique offer advantageous identification and differentiation of the stems cells compared to the conventional FFF and FACS approaches [100].

### 3.4. Drug Delivery

Drug absorption by cells is determined by the drug’s physicochemical properties and formulation. The smaller the particles size is, the easier the drugs are absorbed in the system. Other factors that influence the absorption rate include the molecule’s lipid solubility, size, degree of ionization, and area of the absorptive surface [103,104,105]. Drugs can be absorbed into the cells by passive diffusion, facilitated diffusion, active transport or pinocytosis. Passive diffusion occurs when the drugs diffuse across a cell membrane from a high concentration to a low concentration—for example, the diffusion of drugs from gastrointestinal fluids to blood. The diffusion rate depends on the concentration gradient [106,107].

Most drugs are weak organic acids or bases, while certain drugs are lipid soluble. The physiochemical properties of the drugs will determine the delivery rate [108]. Facilitated diffusion occurs when the drug molecules with low lipid solubility penetrate the cell membrane by forming a carrier-substrate complex and diffusing into the cells. On the other hand, active transport requires energy to transport the drugs into the cell. Active transport is selective and may involve the transport of drugs against the concentration gradient—for example, from a low concentration side to a high concentration side [109]. Active transport usually occurs with endogenous substances such as vitamins, sugars and amino acids. Pinocytosis occurs rarely except for protein-based drugs. During pinocytosis, the cell membrane invaginates and encloses the fluid or particles and then fuses again, forming a vesicle that later detaches and moves to the cell interior [110].

The DEP technique has proven to be beneficial in drug delivery assessment and analysis. Table 5 summarizes the recent drug delivery investigations that were conducted utilizing DEP. Although DEP has brought various advantages to the drug delivery research, DEP has yet to gain recognition by drug delivery researchers.

DEP has been used as a non-invasive diagnostic technique to assess the treatment of the drug delivery. With DEP, the conductivity and permittivity of the membrane and cytoplasm can be determined by the electrophysiological activity of the cells. As previously discussed in the cell membrane subtopic, the ionic imbalance creates a gradient potential, causing the cell membrane capacitance change and affecting the efficacy of the drug delivery into the cells. In drug delivery research, the DEP technique has been used to assess the drug efficacy, cell viability and drug perfusion into the cells. DEP can be applied as a new in vivo technique to monitor drug toxicity, delivery, development, and screening at the single–cell level [74,113,116].

DEP technique has been utilized to observe the drug treatment interaction in the cell by changing the cell membrane capacitance [111]. Furthermore, the microelectrode arrays in [112] have generated weak electro-thermal vortices that support efficient drug mixing and rapid cell immobilization. With this, drug treatment responses can be directly visualize and quantify to provide useful real-time analysis and quantification of the programmed and accidental cell death. As for drug screening, planar interdigitated ring electrode (PIRE) DEP electrode arrays have been used to customize uniform cell patterning and stable drug perfusion. The screening results were equivalent to conventional method; however, with the DEP technique only small amount of cells are required to perform the test; giving DEP a lead for clinical practices especially when there is limited supply of cells [113].

The macromolecular, non-polar and high molecular weight structure of drugs have been found challenging in drug delivery system studies. Despite that, with the iontophoretic DEP electrode, the macromolecule drugs, such as insulin and terbinafine, have changed the membrane conductivity and enhance the drug transdermal delivery [114]. The DEP technique has been also use in concentrating the drug element by entrapping the microparticles and cells within parallel electrode DEP cages.

### 3.5. Viruses

Viruses are very small organisms at the nanometer size. It is the simplest form of organisms because its structure only carries its genetic material. Most viruses or virions (virus particles) have three main parts—nucleic acid, capsid and envelope. Depending on the type of virus, viruses carry their genetic materials as RNA or DNA because the function of the capsid or protein is to cover and protect the nucleic acid. The envelope structure is only present on certain viruses to protect the capsid [117,118,119].

Viruses reproduce inside the host cells. Typically, viruses insert their genetic material into host cells and start replicating [120]. When the host cells lyse, the virus infects other cells and eventually invade the host organ or system. Some viruses may remain inactive inside host cells for long periods with no obvious change in their host cells; however, when the condition becomes favourable, the recurrence infection may occur again [121]. Table 6 lists the DEP investigations on viruses.

Because viruses are small in size, some acute viral infections may cause asymptomatic conditions [129,130]. Furthermore, samples with low virus concentration may lead to false-negative results. This situation has been a large concern to scientists and researchers. There are many immunological techniques and assays with sensitive detectors for virus detection such as the radioimmunoassay (RIA), flow injection analysis (FIA), enzyme immunoassay (EIA) and enzyme-linked fluorescence assay (ELFA) [131,132,133,134]. Despite all of these conventional expensive, high-skills handler diagnostic methods, DEP has shown promising results in detecting, concentrating and discriminating between virally-infected and healthy cells by measuring the electrical properties of the cells.

Recently, researchers have started trapping and detecting viruses using dielectrophoretic impedance measurement (DEPIM) method [122,127]. By varying the electrical conductivity of the suspension liquid and the electric field frequency the number of the trapped viruses is changing. The alteration in the DEP impedance reflected the number of the trapped viruses. Moreover, an optimized gradient insulator-based DEP (g-iDEP) device was utilized for the concentration of particles [123]. The sawtooth design of the g-iDEP device is aimed to selectively capture a variety of bioparticles at different locations in the channel. Additionally, the method described in [124] applied DEP and electrostatic forces on deposited single-walled carbon nanotubes (SWCNTs) to be used as immunosensors. The measurements were taken as the normalized increase in resistance of the immunosensor upon exposure to the viruses. In [125] the researcher have used three dimensional iDEP to increase the viral sequences in order to enhance the probability of detection (virus enrichment). The iDEP force, generated by adjusting the conductivity of the solution and the frequency of the voltage, facilitated the effective transport of the virus without crossing an electrode. In [126], the hydrogel coated microelectrode was utilized to isolate nanoscale particles to high and low electric field gradient regions.

Recently, researchers have used microarray dot electrodes to discriminate dengue-infected cells [18]. The cells’ dielectric properties were quantified by analyzing the light intensity shift within the electrode’s dot region based on the Cumulative Modal Intensity Shift image analysis technique. A unique DEP spectrum was plotted for each normal and virally-infected cells over a wide range of frequencies. The main advantage of this technique is that detecting the viruses occurs indirectly by exploiting the differences in DEP responses occurred to the infected cells rather than detected the small viruses directly. This technique avoids using complex labeling and imaging techniques.

### 3.6. Bacteria

Bacteria are considered eukaryotic cells. The bacterial structure can be spherical, helical, cocci- or rod-like shape, while the arrangement can be in a cluster, chain like, diplococci or mono cocci. Bacteria usually do not cause infection unless the condition favours the optimistic overgrowth of bacteria.

Bacterial infection can be differentiated from viral infection with the white blood cells differential counts [135]. Usually when a patient is having a bacterial infection, the neutrophils counts are higher than the lymphocytes count and vice versa [136]. Regarding the identification process using bacteria, conventionally, it takes a longer time because bacteria need to be cultured first before the identification process can be conducted. For some bacteria such as *Mycobacterium tuberculosis,* it takes a minimum of approximately two weeks to culture the bacteria [137]. There are few studies that have employed the DEP technique for bacterial identification, separation and purification. Table 7 shows the summary of previous DEP applications on bacteria.

Conventionally, there a several methods available for bacterial identification such as staining, chromogenic agar, differential media, enzyme-based reaction or biochemical reaction methods for example indole, motility, Voges Proskauer (VP), methyl red, and citrate, matrix-assisted laser desorption ionization-time-of-flight (MALDI-TOF) mass spectrometry, enzyme linked immunoassay (ELISA) and polymerase chain reaction (PCR) methods [144,145,146,147,148,149,150,151]. These methods have been practiced in many pathology laboratories from local to central and specialized laboratories. Unfortunately, there are many drawbacks of these methods; for the traditional methods such as staining, chromogenic agar and differential media techniques, all of these techniques need proper bacterial culture, a process that is time-consuming and laborious. This will delay the process for the medication prescription and increases the patient’s life risk. On the other hand, techniques such as PCR, MALDI-TOF and ELISA are time effective, accurate and sensitive; nonetheless these techniques are expensive, and only certain laboratories can operate it. These impediments of bacterial identification and research can be overcome by DEP diagnosing techniques, which are cost effective, accurate and time saving.

The method conducted in [138] is based on g-iDEP microchannel which separates similar bacteria strains of a single species. The separations were based on the characteristic of electrokinetic properties based on local electric field strength measurements. While in [142], the researchers have combined the DEP technique with Raman spectroscopy due to low bacteria concentration in the sample. A quadrupole electrode design was used to create the dielectric force. In [140], three dimensional carbon-based electrode have been used to separate intact cells from damaged cells. The total intensity of trapped cells around the electrodes was used in the quantification measurements. Moreover, a flow-cell device was constructed to evaluate DEP separation of bacteria and clay in a continuous flow through mode [141]. In [142], a rapid antibiotic susceptibility test (AST) was done based on the changes in DEP behavior related to the bacteria. The inhibition of the antibiotic was measured by the positive DEP frequency response and the length of the bacteria. In addition, the interdigitated DEP electrodes combine with the impedance analyzer were used to measure the bacteria concentration [143]. As the trapped bacteria concentration increases, the impedance decreases.

### 3.7. Mycoses

Mycoses are fungal infections that can infect humans or animals. Fungi can be eukaryotic cells or prokaryotic cells, while the structure can be filamentous or budding. Certain multicellular complex fungi can have a mushroom shape. Fungi favour humid and dark conditions to grow. Fungal infections can cause severe illnesses, especially when they enter the human internal system. For example, fungi such as *Aspergillus* can grow in the human lung and causes severe toxicity and allergy, while *Cryptococcus* can cause meningitis and brain damage in autoimmune patients [152,153].

Fungal detection is almost the same as that of bacteria—they require their own special staining, media and biochemical reactions [154,155,156,157,158]. Fungal cultures are also time consuming and laborious [159]. Tang et al. [160] used *S. cerevisiae*, a type of yeast, to investigate the DEP response of lyticase (cell lysis agent). The research was used as a modular platform of the cellular response subjected to apoptosis chemical stimulation as well as physical stimulations down to the single-cell level. However, Patel et al. [161] has also used yeast as a platform to check the cell viability using reservoir-based DEP. Although the fungal infection may cause severe infection in humans, there has been only limited research to exploit the advantages of the DEP technique to diagnose mycoses.

### 3.8. DNA

Deoxyribonucleic acid (DNA) is built on the simpler units of the nucleotides cytosine, guanine, thymine, and adenine with a deoxyribose sugar and a phosphate group. DNA stores biological information and carries the genetic materials for growth, development, body function and reproduction of all known living organisms and some viruses. DNA can be found in the nucleus and in the mitochondria. DNA research is vital because it can be implemented in gene therapies for inherited disease research, genetic enhancement and even in legal aspects such as forensic and paternity claims [162,163,164]. There are several DNA studies conducted using the DEP technique mainly due to its advantages in the separation and isolation of small particles (see Table 8).

DNA sample preparation is laborious, tedious and requires meticulous attention during the process. The steps during the preparations, such as pipetting, centrifuging and filtrating, would sometimes degrade the DNA quality and quantity [166,169,172]. The purification, recovery and isolation from whole-blood samples would also be a major challenge in DNA research [166,167,169]. Furthermore, a common electroporation instrument such as an optical tweezer will damage the cell membrane during the transfection [165]. DEP techniques have circumvented the research impediment by providing a high resolution of isolation and separation of target bio-particles directly from the whole blood and other biological fluid samples [126,173,174,175]. Furthermore, unlike optical tweezers, DEP provides a low applied voltage and an alternating current for gene transfection from DNA that can prevent cell membrane damage [165]. DEP techniques use a smaller sample volume and can isolate to the extent of a nanoscale-sized bio-particle, making it an excellent candidate for further development as a point-of-care (POC) diagnostic tool, especially in molecular research [176,177,178].

DEP-assisted electroporation was developed by using light-activated virtual microelectrodes in a microfluidic platform [165]. The electrotransfection have used a low applied AC voltage to enable electroporation and transfection. Moreover, researchers have implemented a DEP-based microarray chip for extracting circulating DNA [166,167,169]. On the other hand, DEP force, generated by two combine electrodes stretching system, was used to immobilized λ DNA molecules [168]. In addition, the DEP technique was successfully used to trap a single DNA molecule with a silicon nanotweezers [170]. In [171], an immunodevice was developed for capturing DNAs by combining microparticle-based immunoreactions with n-DEP accumulation and trapping.

### 3.9. Proteins

Amino acids are the building blocks of proteins. Other than being important for building muscle and bone, proteins are used as hormones (e.g., dopamine), oxygen transport (transferrin) and enzymes (all enzymes are proteins) [179,180]. Although the genetic materials are stored as DNA, all of the processes of DNA replication and cell replication such as mitosis and meiosis are based on proteins [181]. On the other hand, our immune system is also based on proteins. It works in our body to differentiate between self and non-self-organisms based on the major histocompatibility protein on the cell surface [182]. These are only a few examples of the uses of protein in the human body.

There are many diseases due to protein deficiencies in the body. For example, deficiencies in protein C and protein S may cause abnormal blood clotting [183]. A protein deficiency disease called cachexia cannot be reversed nutritionally. Patients who have cachexia face muscle atrophy, weakness and extreme fatigue [184].

Despite the important functions of proteins in the body, there are only limited studies on the DEP technique that focuses on proteins compared with other fields. Nakano and Ros have extensively reviewed DEP applications in proteins [185]. They have emphasized that the DEP technique plays an important role as a manipulation, fractionation, concentration, and separation method in protein research. On the other hand, they indicated that protein immobilization is an obstacle in DEP due to Brownian diffusion, whereas other bulk fluid flows such as electroosmosis overpower DEP. However, in 2015 this problem has been resolved by Laux et al. by using higher spatial atomic force that enables the visualization of the protein distribution on single nanoelectrodes [42].

Enzymes are proteins that act as catalysts to accelerate biological reactions. They are temperature sensitive and very specific. Similar to proteins, there is also limited research on enzymes. In 2009, Baret et al. have proven that the DEP technique is efficient in cell sorting based on β-galactosidase enzyme activity using a fluorescence-activated droplet sorter (FADS) and traditional fluorescence-activated cell sorting (FACS) [186]. Furthermore, Laux et al. immobilized horseradish peroxidase enzyme molecules while retaining their activity and rendering any chemical modifications unnecessary by the DEP technique [187].

## 4. Sensitivity and Specificity

Powerful clinical diagnosing tools must comprise of a combination of a high levels of sensitivity and stringent specificity to secure a true positive results in disease identifications. Signatures identifications and isolations of bio-particles have become major impediments in diagnosing the ambiguous diseases symptoms. Diagnostic results accuracy along with the speed identifications of bio particles is critical in timely infection and fast communicable diseases. The stat test and long turnaround time especially in using cultures in conventional methods of identification takes ages and the patient’s life is endangered. Moreover, some diseases for an example cancer can only be identified in the advance stage, making it difficult for the treatment [188].

In addition to all the advantages of DEP techniques discussed earlier in each application, DEP techniques assist and improve the clinical sample sensitivity in detection and separation of target bio- particles [189,190]. Currently, the normal standard of dengue infection detection is by using protein expression of antibody and antigen such as ELISA, Haemaaggluitination Inhibition (HAI) and reverse transcriptase- polymerase chain reaction (RT-PCR) techniques [191,192,193,194,195]. However, recently the DEP techniques have shown to be promising diagnostic tools by manipulating the dielectric properties of the cell cytopathic effect in infection [18,196]. Conversely, the DEP techniques also provide a great advantages by preserving the correlation of specificity and sensitivity forcing the compromise of high-throughput and highly specific isolation of bio particles label free applications with minimal sample preparations [197]. The high specific and specificity identification of target bio- particles from complex biological fluid such as blood [198] and urine [199] can be used as a solid proof that DEP methods have broad potential as powerful diagnostic tools.

## 5. Current Challenges and Limitations

DEP has been demonstrated to have the potential to be the most convenient assistive POC diagnostic technique that can aid in the screening and identification of diseases. In biomedical sciences research, it helps to isolate, trap, separate, fractionate, and concentrate target bioparticles for many research uses. In addition to the DEP techniques being label-free, they are cost- and time-effective techniques.

However, when proposing a new technology, there is a long and winding road before it enjoys worldwide recognition. There are many obstacles faced by scientists to develop the best DEP electrode to meet the research need. Some researchers face problems such as bubble formation in liquid, which affects electrical insulation, darkening of the electrode under high conductance conditions in DC, and some face problems with microchannels due to high gradients acting only in the vicinity of the electrodes [165,185,200]. Joule heating also becomes a challenge to researchers. Kale, reported that Joule heating reduces the reservoir DEP (rDEP) focusing and trapping performance due to the rise of fluid temperature and reduces the electric field at the reservoir-microchannel junction [201]. Furthermore, it was reported that the cell viability can be significantly decreased after iDEP manipulation mainly due to direct damage to the cell membrane caused by the electric field combined with joule heating [202].

Beside all of the limitations above, there are also many things that need to be considered in DEP techniques such as the evaporation of water or liquid during DEP experiments, which may cause variations in concentrations and osmolarity [112]. The suspending media used also need to be considered in DEP experiments. The CM factor K(ω) can be positive or negative depending on the relative polarizability between the cell and suspending medium, creating the pDEP and nDEP effect. This can be controlled by selecting the appropriate frequency of the applied electric field [18]. In most DEP experiments, the typical media used in cell culture techniques (i.e., phosphate-buffered saline (PBS); Dulbecco’s Modified Eagle Medium (DMEM)) cannot be used in DEP experiments due to their high conductivity, and a low-conductivity medium or buffer is usually used as the suspending medium; however, the medium was reported to have a notable influence in decreasing cell viability after 6 h of incubation [112].

Although the DEP analytical techniques are economically wise, the first thing that needs to be considered is the user—for instance, the doctors, the clinical laboratory scientists, the medical researchers and the laboratory technicians. It is hard to change the people personnel’s paradigm that has been well niched with the conventional diagnostic methods. The DEP technique needs to be educated, promoted and demonstrated—training and a seminar should be provided to these users so they will gain confidence in using it.

## 6. Recommendations and Potential Applications

DEP is an accurate, label-free and rapid technique for cell sorting, differentiation, trapping and purification. Furthermore, the DEP technique has the potential to unbound the research laboratories from the bulky and complex machines; meanwhile, it does not require highly trained personnel for operation.

Although many research experiments have been conducted for various types of biological samples using the DEP techniques, reported investigations are still at the teething phase. These studies were mainly focused on understanding the fundamental response of biological particles to DEP forces. Few studies have attempted to link the DEP behaviours of the biological particles to their electrophysiological properties. However, there is still a high demand to integrate the DEP techniques with miniaturized lab-on-a-chip platforms to perform various bio-research applications.

A complete DEP system should consist of several essential components, including a microelectrode device, a signal generator, an image-capturing device, autograph analysis and computer software. Such a system would be able to conduct sample preparation, detection and robust signal quantification automatically, leading to a complete convenient functional system. The engineers should have developed a complete system with universal cell suspending solutions, image and graph analysis with a standard reference database to make DEP techniques user friendly by the biomedical researchers.

DEP technique can be a potential assistive tool to distinguish between normal and damaged cells of fibroblasts in antiaging projects. The delivery and effectiveness of anti-aging plant extract or compound in the cells can be assessed by the unique DEP responses of respected cells. The cells that are damaged by UVA and UVB can be characterized by DEP since each type of UV will have a different effect on the cells’ electrophysical properties. Moreover, DEP can also be implemented as a treatment assessor in the dermatology industry. With the use of DEP, the cost of dermatology product testing would be reduced, and the use of animal testing in dermatology product development can be stopped.

Since the beginning the DEP field, metal-based electrodes (i.e., nickel, gold, platinum, silver, etc.) have been used to generate the non-uniform electric field needed for DEP. Planar electrodes have been widely used to induce DEP effects. In order to have stronger electric fields, and thus stronger DEP responses, a few 3D microelectrodes were proposed in the literature. However, these microelectrodes were fabricated via complex processes using expensive machineries. Alternatively, 3D DEP electrodes may be fabricated using polymer precursors before pyrolyzing them in an inert atmosphere to become carbon [203]. Carbon has a number of advantages including a wider electrochemical stability window compared to noble metals. This will reduces the possibility of sample electrolysis for a given applied voltage. Furthermore, carbon enjoys excellent chemical inertness and biocompatibility.

## 7. Summary

Medical sciences are an integrated multidisciplinary field that involves the study of the mechanisms of life and underlying causes of disease, and they seek to develop, improve and search for the unmet treatments and diagnosis for patient populations. DEP is one of the promising techniques that can meet biomedical research needs. Researchers need to direct their efforts toward the development of POC devices to be used where central laboratories are inaccessible—for example, in villages and developing countries. These POC devices would allow the diagnosis to be made as early as possible to save a patient’s life and deliver the right treatment. The DEP technique would be a big helping hand to untie the tangled issues of the increasing global cost of clinical laboratory tests.

This paper has reviewed research activities conducted in the biomedical sciences utilizing DEP, including studies on eukaryotes, prokaryotes, cell membranes, oncology research, stem cells, drug delivery, viruses, bacteria, mycoses, DNA, proteins and enzymes. Then, a brief outline of the current challenges that DEP research faces was presented. Finally, future potential directions of DEP in the biomedical sciences research arena were proposed.

In this review, we conclude that DEP is a powerful, cost-effective, fast, accurate and label-free analytical diagnostic and screening technique. It has been demonstrated that DEP techniques offer many advantages in isolating, trapping, separating, concentrating, and fractionating bioparticles down to the nanoscale dimension. With this paper, it is hoped that the frontier gap between biomedical sciences and engineering can be closed and progress to create better technology and improve human health. It is expected that the DEP technique can help to change medical and public-health scenarios, especially in developing countries, using new dynamic analytical screening and diagnostic tools.

## Figures and Tables

**Table 1:** Electrode geometries and arrangements used in DEP for biomedical applications.

Electrode Geometry	Applications	Advantages	References
Electrodeless Insulator-based DEP (iDEP)	Particle trapping of nanoscale bio particles	High vast localized electric field gradient	[36,37,38,39]
Rectangular electrode	Determination of nanoparticles concentration	Manipulate particle spacing to observe various particle conditions	[40]
	Liquid pumping manipulation in microchannel electrode	Obviate pumping and leakage problems in close channel	[41]
Cylindrical electrodes	Immobilization of protein molecules	Label free protein molecule quantification	[42]
	Single cells characterization manipulation	Antiparallel DEP field	[43]
Interdigitated electrode	Cell differentiation	Cell characterization based on bio-electrical properties	[44]
	Nanoparticles quantification	Real time image quantification method of nanoparticles	[45]
	Particle motion prediction	High throughput and low energy consumption	[46]
Circular electrode	Infected cell discrimination	Simple results’ interpretation by crossover frequencies	[18]
	Particle separation	Low volume of sample	[47]

**Table 2:** DEP investigations of eukaryotic and prokaryotic cells.

Type of Cell	Applications	Advantages	References
Eukaryotes: Cancer cells Erythrocytes HeLa	Sorting and trapping	More efficient cell sorting and trapping	[66]
Prokaryotes: Bacteria (*Escherichia coli*)	Separation	Improve assay sensitivity	[36]
Bacteria (*lactobacillus*) and yeast	Separation	Independent fingerprinting and label-free separation of microbes	[67]
Bacteria (*Clostridium*)	Trapping		[68]

**Table 3:** DEP investigations in oncology.

Cell Type	Applications	Advantages	References
CTCs	Isolating CTCs from blood	Rapid and label-free cell isolation method	[79]
Human oral cancer cells	Cancer cell characterization	Rapid and label-free cells characterization method	[80]
Osteosarcoma (Bone cancer cells)	Identification and monitoring of tumour heterogeneity	Label-free cancer subset characterization	[81]
Breast and colorectal cancer	Differentiations of two cancer cells	Label-free isolation and separation of cells	[19]
Prostate cancer	Rare cancer cell isolation from blood	Improve immunocapture performance	[82]

**Table 4:** DEP studies on stem cells.

Applications	Details	References
Stem cells differentiation	Neural stem and progenitor cells with more neurogenic progenitors (NPs) can be distinguished from those with more astrogenic progenitors.	[96]
	Human mesenchymal stem cells (hMSCs) and their differentiation progenies (osteoblasts) by multiple DEP force.	[97]
	Mouse embryonic stem cells (mESCs) and C2C12 skeletal muscle myoblasts. DEP spatially organize the cells and their spheroids.	[98]
Cell fractionation	Adipose tissue stem cells fractionated in a suspension using DEP field flow.	[99]
Cells isolation and sorting	Isolation of mouse neural stem/precursor cells (NSPCs) to the progenitor cells with different dielectric properties by DEP.	[100]

**Table 5:** DEP investigations in drug delivery research.

Drug name	Applications	Details	References
Gefitinib (ZD1839)	Cancer treatment	Drug treatment assessments	[111]
Cycloheximide (CHX)	Protein biosynthesis inhibitor	Cells immobilization	[112]
Cisplatin and docetaxel	Chemotherapy drug	Drug screening	[113]
Terbinafine and insulin	Anti-fungal and diabetic treatment	Drug delivery enhancements	[114]
Lipospheres	Particle for coating drugs for oral administrations	Concentrating the drugs	[115]

**Table 6:** DEP investigations on viruses.

Virus Type	Virus Size	Diseases	Applications	References
Adenovirus	90–100 nm	Respiratory disease	Virus detection and trapping	[122]
Rotavirus	80 nm	Gastrointestinal disease and inflammation	Virus detection and trapping	[122]
Sindbis virus	60 nm	Sindbis fever (Similar to chikungunya fever)	Isolation, detection and concentrating the viruses	[123]
H1N1	80–120 nm	Viral influenza	Virus detection	[124]
The influenza viruses (A PR/8)	80–120 nm	Viral influenza	Virus enrichment	[125]
T7 bacteriophage virus	60–61 nm	Invade the bacteria	Virus isolation	[126]
Norovirus	26–35 nm	Gastrointestinal disease and inflammation	Virus trapping	[127]
Dengue	40–60 nm	Dengue fever	Virus discrimination	[18]
HIV	120 nm	AIDS	Virus detection	[128]

**Table 7:** DEP investigations of bacteria.

Bacteria	Applications	References
*E. coli*	Identification and separation of bacteria	[138]
*E. coli* and *Enterococcus faecalis*	Pathogen specification and separation	[139]
*Mycobacterium smegmatis*	Separation of cells	[140]
*B. atrophaeus*	Separation of soil particle and bacteria	[141]
*E. coli* and *Klebsiella pneumonia*	Reduction of the bacterial growth time and drug sensitivity assay	[142]
*E. coli*	Measurements of bacterial concentrations in a medium	[143]

**Table 8:** DEP investigations of DNA.

Applications	References
DNA transfection	[165]
Rapid discovery of circulating cell free DNA from plasma	[166]
Direct detection of DNA from whole blood	[167]
Manipulation and characterization to immobilized *λ* DNA	[168]
Rapid, simple, and label free cancer cell-free DNA isolation	[169]
Stretching and trapping DNA single-DNA molecule	[170]
Sensitive, rapid and simple DNA trapping for particle manipulation	[171]
